# The Case for Live Attenuated Vaccines against the Neglected Zoonotic Diseases Brucellosis and Bovine Tuberculosis

**DOI:** 10.1371/journal.pntd.0004572

**Published:** 2016-08-18

**Authors:** Aseem Pandey, Ana Cabello, Lavoisier Akoolo, Allison Rice-Ficht, Angela Arenas-Gamboa, David McMurray, Thomas A. Ficht, Paul de Figueiredo

**Affiliations:** 1 Department of Microbial Pathogenesis and Immunology, Texas A&M Health Science Center, Bryan, Texas, United States of America; 2 Department of Veterinary Pathobiology, Texas A&M University, College Station, Texas, United States of America; 3 Norman Borlaug Center, Texas A&M University, College Station, Texas, United States of America; 4 Department of Molecular and Cellular Medicine, Texas A&M Health Science Center, Bryan, Texas, United States of America; University of California San Diego School of Medicine, UNITED STATES

## Abstract

Vaccination of humans and animals with live attenuated organisms has proven to be an effective means of combatting some important infectious diseases. In fact, the 20th century witnessed tremendous improvements in human and animal health worldwide as a consequence of large-scale vaccination programs with live attenuated vaccines (LAVs). Here, we use the neglected zoonotic diseases brucellosis and bovine tuberculosis (BTb) caused by *Brucella* spp. and *Mycobacterium bovis* (*M*. *bovis)*, respectively, as comparative models to outline the merits of LAV platforms with emphasis on molecular strategies that have been pursued to generate LAVs with enhanced vaccine safety and efficacy profiles. Finally, we discuss the prospects of LAV platforms in the fight against brucellosis and BTb and outline new avenues for future research towards developing effective vaccines using LAV platforms.

## Background

Vaccination provides the most effective means of preventing and eradicating infectious diseases, and historically, live attenuated vaccines (LAVs) have proven effective in protecting humans and animals from infection. LAVs are weakened versions of the pathogen, obtained by disrupting or mutating one or more genes, which renders the pathogen incapable of causing disease; however, the pathogen remains sufficiently potent to educate the immune system to initiate and establish highly specific short-term or lifelong immunity [1]. LAVs offer a promising approach because they fail to induce disease in vaccinated individuals while simultaneously mimicking natural properties of the virulent organism, including cell invasion and tissue tropism [2], and presentation of a broad repertoire of antigens [3]. Moreover, LAVs have been successfully used as vaccines for several human diseases of bacterial or viral origin [4–7]. Importantly, for some diseases, including brucellosis and bovine tuberculosis (BTb), LAVs have demonstrated greater efficacy and/or safety in various model systems than competing subunit vaccine platforms (Table 1) [8–12]. Therefore, LAVs have demonstrated safety and efficacy in human and animal populations and provide an attractive strategy for combatting neglected zoonotic diseases. Here, we provide a broad overview of LAV technology, describe recent progress in the development of this approach for neglected diseases, and outline challenges that must be addressed to develop vaccines with broad safety and efficacy profiles. We use a comparative model to frame our discussion of these issues, and focus on brucellosis and BTb, which have been designated as neglected zoonotic diseases by the World Health Organization [13,14].

**Table 1:** Live attenuated vaccines.

Strain Name or Gene Deleted	Species Tested in	Challenge Strain/Dose/Route	Efficacy/Protection against Abortion	Vaccination Dose/Route	USDA Approval Status/ Comments	References
***Brucell*a spp.**						
***B*. *abortus***						
*∆bp26*	Cattle	B.a (2308) 3 × 10^8^ (SC)	81%	1 × 10^10^ (SC)	Experimental	[15]
*∆p39*	Mice	B.a 2 × 10^5^ (IP)	99%	1 × 10^5^ (SC)	Experimental	[16]
*∆pgk*	Mice	B.a 1 × 10^5^ (IP)	99%	1 × 10^5^ (IP)	Experimental	[17]
*∆pgm*	Mice	B.a (2308) 5 × 10^5^ (SC)	99%	1 × 10^7^ (IP)	Experimental	[18]
*RB51*	Cattle	B.a (2308) 1.5 × 10^10^ (IC)	100%	3 × 10^8^ (SC)	Approved for animal use	[19]
*S19*	Bison	B.a (2308) 1 × 10^7^ (IC)	67%	5.3 × 10^6^ (SC)	Approved for veterinary use	[20]
*S19*	Cattle	B.a (2308) 9 × 10^4^ (IC)	70%–91%	1 × 10^9^ (SC)	Approved for veterinary use	[21]
*∆vjbR*	Mice	B.a 1 × 10^5^ (IP)	99%	1 × 10^5^ (IP)	Experimental	[22]
*∆wbkC*	Mice	B.a 1 × 10^6^ (SC)	73%	1 × 10^8 (^IP)	Experimental	[23]
*∆znuA*	Mice	B.a 5 × 10^4^ (SC)	90%	1 × 10^8^ (IP)	Experimental	[24]
***B*. *melitensis***						
*∆asp24*	Goat	B.m 1 × 10^7^ (IC)	62%	1 × 10^6^ (IP)	Experimental	[25]
*∆bp26*	Sheep	B.o 1.7 × 10^9^ (IPre)	100%	1 × 10^9^ (SC)	Experimental	[26]
*∆bp26* and *∆omp31*	Sheep	B.o 1.7 × 10^9^ (IPre)	84%	1 × 10^9^ (SC)	Experimental	[26]
*∆htrA*	Goats	B.m 1 × 10^7^ (IC)	100%	1 × 10^9^ (SC)	Experimental	[27]
*∆manBA*	Mice	B.m 1 × 10^4^ (IC)	87%	1 × 10^6^ (IP)	Experimental	[25]
*∆mucR*	Mice	B.m 5 × 10^9^ (IN)	99%	1 × 10^6^ (IP)	Experimental	[28]
*∆omp25*	Goats	B.m 1 × 10^7^ (IC)	100%	1 × 10^9^ (SC)	Experimental	[29]
*∆per*	Sheep	B.m 4.9 × 10^7^ (IM)	36%	1 × 10^10^ (SC)	Experimental	[30]
*∆purEK*	Mice	B.m 1 × 10^4^ (IN)	99%	1 × 10^11^ (oral)	Experimental	[31]
*Rev 1*	Goats	B.m 16M 1.25 × 10^6^ (SC)	100%	1.5 × 10^6^ (SC)	Approved for animal use	[32]
*∆virB2*	Goats	B.m 1 × 10^7^ (SC)	75%	1 × 10^9^ (SC)	Experimental	[25]
*∆virB2*	Goat	B.m 1 × 10^7^ (IC)	75%	1 × 10^9^ (SC)	Experimental	[25]
*∆vjbR*	Mice	B.m 16M 1 × 10^5^ (IP)	60%	1 × 10^5^ (IP)	Experimental	[33]
*∆wa*	Sheep	B.m 4.9 × 10^7^ (IC)	31%	1 × 10^10^ (SC)	Experimental	[30]
*∆wbkF*	Mice	B.m 4.9 × 10^7^ (IC)	54%	1 × 10^10^ (SC)	Experimental	[30]
*∆wboA*	Goat	B.m 1 × 10^7^ (IC)	20%	1 × 10^9^ (SC)	Experimental	[34]
***B*. *ovis***						
*ΔabcBA*	Rams	B.o 3.6 × 10^9^ (IPre/IC)	100%	1 × 10^9^ (SC)	Experimental	[35]
***M*. *bovis (BCG)***						
*BCG*	Cattle	M.b 1 × 10^3^ (Aer)	64%	1 × 10^3^ (Aer)	Approved for animal use	[36]
*∆LeuD*	Cattle	M.b 1 × 10^6^ (SC)	60%	1 × 106 (IN)	Experimental	[37]
*∆mce2*	Cattle	M.b 1 × 10^6^ (SC)	80%–90%	1 × 10^6^ (IT)	Experimental	[38]
*∆p27-p55*	Mice	M.b 1 × 10^5^ (SC)	96%	1.25 × 10^6^ (IT)	Experimental	[39]
*RD1*	Cattle	M.b 1 × 10^3^ (Aer)	80%	1 × 10^3^ (Aer)	Experimental	[36]

Abbreviations: Aer, Aerosol; B.a, *Brucella abortus*; BCG, Bacillus Calmette–Guérin; B.m, *B*. *melitensis;* B.o, *B*. *ovis*; IC, intraconjunctival; IM, intramuscular; IN, intranasal; IP, intraperitoneal, IPre, intrapreputial; IT, intratracheal; M.b, *Mycobacterium bovis*; SC, subcutaneous; USDA, US Department of Agriculture

Brucellosis, also called Bang’s disease, is a worldwide zoonosis of profound importance [40]. Globally, half a million people develop infection each year [40], and millions of livestock are either infected or potentially at risk of acquiring infection [41]. Despite these reports, the actual numbers are suspected to be 2–5 times higher because of chronic under-reporting of infection and misdiagnosis [40]. Although brucellosis prevalence is low in most of the Western world, it is endemic in human and livestock populations in Asia, South and Central America, and sub-Saharan Africa [42–47] and affects not only the health but also the livelihood of people who rely upon livestock-related economic activities [47].

In humans, brucellosis typically presents as an undulant fever but gradually becomes systemic, affecting practically every organ system of the body with protracted symptoms of arthopathy, myalgia, and debilitation [48]. In ruminants, the reproductive system is a common site of infection, and the disease during gestation increases the risk of spontaneous abortion and human exposure [49]. Brucellosis has eluded systematic attempts at eradication for more than a century [50], even in most developed countries, and so far no vaccine is approved for human use [3]. Human brucellosis is associated with low rates of mortality and high rates of morbidity, and hence, *Brucella* has the potential to render patients severely debilitated, which can perpetuate poverty and tax health care resources [51,52]. Although combination antibiotic therapy can be used to treat *Brucella* infection, typical treatment regimens are prolonged and are often accompanied by unwanted side effects [53].

The intracellular bacteria *Mycobacterium bovis* induces both pulmonary and extrapulmonary symptoms in humans and animals [54]. *M*. *bovis* shares 99.95% genome sequence similarity with *M*. *tuberculosis* and a live attenuated version of the pathogen, Bacillus Calmette–Guérin (BCG), provides the current and only widely used vaccine against tuberculosis [55]. The global incidence of tuberculosis caused by *M*. *bovis* in humans as well as a wide variety of animal species is increasing. More than 50 million cattle are estimated to be infected with the pathogen worldwide [56], resulting in more than US$3 billion in agricultural losses annually [57]. *M*. *bovis* infection of humans presents with similar symptoms as *M*. *tuberculosis*; however, human infection with virulent *M*. *bovis* infection is not responsive to the antibiotic treatment commonly used to treat *M*. *tuberculosis* [58]. Although BTb has been eradicated from most of the developed world through regular tuberculin testing and culling of infected livestock [59], wildlife reservoirs of the causative agent constitute a significant global veterinary and public health threat [60], especially in resource-poor regions where culling is neither affordable nor practical [59].

The pathogenic programs of *Brucella* and *M*. *bovis* share similar features that support LAV development. First, the unavailability of highly effective subunit vaccines that protect humans against these pathogens can be largely attributed to their intricate immune evasion strategies [61–67]. However, recent advances in vaccinology suggest that improved LAVs for animal (and eventually human) use against these diseases may be within striking distance [3]. Therefore, these organisms provide a useful comparative model for considering progress in LAV development. Second, *Brucella* spp. and *M*. *bovis* are intracellular vacuolar pathogens that establish replicative niches within acidic compartments of professional phagocytic cells [68] and therefore have evolved mechanisms to subvert host factors [69] including conserved innate immune defenses [70], phagosome maturation [67] and phagolysosome acidification [68]. Therefore, vaccines that activate macrophage-mediated killing of resident pathogens [71] or stimulate activation of cytotoxic T lymphocytes that kill infected cells are desirable. Finally, gaining a better understanding of interactions between *M*. *bovis* and *Brucella* spp. and their respective host proteins will reveal novel avenues for engineering next-generation LAVs. The purpose of this article is not to comprehensively review the development of vaccines for *M*. *bovis* or *Brucella* spp., which can be found elsewhere [72–76], but rather to use a comparative approach with vaccines directed against these pathogens to elucidate the utility of LAVs for neglected zoonotic bacterial diseases.

## Early Development and Use of LAVs

Several approaches to LAV generation have been described (Table 2), including serial passage of the virulent organism [77], use of nonhost species for vaccination, exposure to varying culture conditions or irradiation [78], or the identification and deletion of genes that contribute to symptomology or disease progression [33]. Historically, attenuation by serial passage has been a preferred approach for LAV generation, and based on this, many important vaccines, including the BCG vaccine against tuberculosis, have been successfully developed [79]. This approach involves multiple cycles of growth of the bacteria under cultivation conditions that ultimately lead to an accumulation of genetic mutations that result in altered virulence. For example, 13 years of serially passaging *M*. *bovis* resulted in attenuation and subsequent establishment of *M*. *bovis* BCG [55]. Serial subculturing has been shown to induce various types of mutations that significantly alter the virulence of the organism, although serial passage can also sometimes induce fitness-increasing mutations that enhance bacterial survival [80]. Strains with attenuated virulence and normal replication rates constitute useful reagents for the development of LAVs against *M*. *bovis*. Serial passage has also been used to generate *Brucella* vaccines strains. For example, RB51 is a spontaneous rough mutant derived using repeated passage of *Brucella abortus* strain 2308 in vitro [81]. The main drawbacks to serial passage as a strategy for deriving LAV strains are that it neither reveals the molecular mechanisms that cause attenuation nor guarantees that a safe and effective vaccine will result from the effort. In fact, some vaccines prepared in this fashion, including *B*. *abortus* strains 45/20 [82], proved to be nonprotective or susceptible to reversion to wild-type virulence. Knowledge of the genetic basis of attenuation is key to understanding the mode of action of the developed vaccines. Hence, other approaches have been pursued that involve prior identification of virulence genes followed by the induction of targeted mutations.

**Table 2:** Approaches to LAV generation.

Approaches	Advantages	Disadvantages
**Multiple Passages, Chemical, Physical, or Nontargeted Mutagenesis**	Contains broad antigenic determinants [83,84]	May induce disease [83,92,93]
	Relatively easy to generate [85]	Genomic loci of mutations may be initially unknown, or genetic instability may be observed [94]
	Induction of humoral and cellular immune responses [86,87]	Risk of acquisition of antibiotic-resistant phenotypes
	Various degrees of durable immunity elicited [88,89]	Difficult to distinguish between animals naturally infected from those immunized [95]
	Adjuvant not required for protective efficacy [22,89]	Antibiotic resistance selectable markers used for generation of mutants may lead to regulatory hurdles
	Loss of virulence factors encoded by extra chromosomal plasmids [90,91]	
**Targeted Gene Deletion**	Expected genetic stability of mutations	Possible recombination events with dormant genes and consequent safety implications
	Reduced risk of reversion [96]	Exchange of genetic information with other vaccine or wild-type strains and consequent safety implications
	Ability to differentiate infected from vaccinated animals (DIVA) [95,97]	
	Loss of pathogenicity [98]	

Exposure of microorganisms to irradiation [78,99] or other conditions, including low temperature [100] and chemicals [101], can be used to induce attenuating chromosomal mutations for the purpose of developing LAVs. *B*. *abortus* strain 19 (S19), for example, is a smooth strain that became attenuated during prolonged cultivation under dehydrating conditions [102]. The molecular basis for the attenuation of S19 is not yet known. However, studies have demonstrated that S19 harbors mutations in 24 virulence-associated genes [103], including genes encoding an outer membrane protein and three proteins involved in erythritol uptake or metabolism [103]. Irradiation can also be used to generate organisms displaying reduced replicative capacity in vivo while preserving metabolic and transcriptional activity [78,104], an ability to persist in macrophages [78], and the capacity to confer protection to mice against virulent bacterial challenge [78]. More recent high-throughput approaches for generating and screening banks of mutant bacteria include the transposon-site hybridization (TraSH) system [105] and RNA-guided gene editing using clustered regularly interspaced short palindromic repeat-CRISPR-associated protein (CRISPR-Cas) technology [106]. The application of these approaches for LAV generation constitutes an exciting area of future investigation.

Transposon-mediated mutagenesis by random gene inactivation has been used to identify virulence factors and construct mutations in *Brucella* [107,108] and *M*. *bovis* [109,110]. This approach in *M*. *bovis* yielded mutants with similar efficacy to BCG in a guinea pig model of tuberculosis [111]. In *Brucella* spp., this strategy led to the mutation and identification of several virulence genes [112]. For example, strains harboring mutations in VjbR, a quorum sensing-related transcriptional regulator [108,113], were demonstrated to be potential vaccine candidates based on significant reductions in virulence revealed in in vitro and in vivo models of *Brucella* infection [22,33,114]. Transcriptomics may also enable the identification of virulence genes. Similarly, protein arrays have been used to identify surface-localized immunogenic proteins [115]. This strategy involves screening protein arrays using sera from vaccinated or infected animals to identify target vaccine antigens. Sera from infected or convalescent patients have also been used to screen protein arrays containing pathogen proteins to characterize rates of infection and identify bacterial antigens [44]. Following identification of loci of interest, targeted gene mutations can be introduced at these loci using conventional bacterial gene targeting approaches [85] or gene editing technology [106]. Finally, the mutated strains can be tested for virulence (i.e., safety) and for the ability to confer protection against challenge with virulent organisms (i.e., efficacy).

## Recent Progress in the Development of New *Brucella* Vaccines

Various vaccine modalities, including DNA, protein, viral vector, and live attenuated vaccines, have been developed for protecting animals or humans from brucellosis [74,76]. For example, several protective antigens for brucellosis, formulated either as DNA or purified proteins, have been tested in murine models under various challenge regimes. These antigens include several outer membrane proteins (OMPs) [116–118], DnaK and SurA [119], and lumazine synthase [120]. Moreover, live vaccine vectors (e.g., *Salmonella enterica* serovar Typhimurium) that express heterologous *Brucella* protective antigens (e.g., L7/L12 and lumazine fusion protein) have been tested [121]. Vaccinia virus [122] or Semliki Forest virus [12,123] vectors have also been used to deliver the *Brucella* vaccine antigens L7/L12 [122], SodC [123], or translation initiation factor [12] with the aim of eliciting protective responses in murine models of *Brucella* challenge. However, only modest protection, which was lower than reported for the LAVs RB51 or S19, was observed in these studies.

While an effective subunit vaccine for *Brucella* has yet to be developed, several LAV formulations show promise. Although efficacy has been demonstrated in bovine populations with S19, this vaccine can induce abortion in pregnant animals [124]. It can also cause disease in humans as a result of secondary exposure and is thus considered to be unsafe for use in humans. The vaccine strain RB51, which is used in cattle, and Rev.1, which is used in sheep and goats, can induce abortion in pregnant animals [75,125] or infect humans [126]. The search for improved LAVs against brucellosis has relied upon advances in our understanding of *Brucella* virulence determinants and the role of individual genes in the survival and virulence of the pathogen in vitro and in vivo. Collectively, this work has provided opportunities to build upon the merits of the LAV approach by using rational bioengineering of strains for the generation of attenuated agents that harbor deletions of key genes that are essential for virulence yet maintain efficacy in vaccine challenge experiments. However, it is often easier said than done with *Brucella* spp., as well as with other bacterial pathogens, which unlike their viral counterparts have larger genomes and greater genetic complexities that require defining strains with levels of attenuation that ensure both safety and protection. Continued efforts in our labs to develop attenuated *Brucella* mutants as vaccine candidates have yielded promising candidates, including *∆mucR* [28], *∆asp24* [127], and *∆vjbR* mutants [33]. *B*. *melitensis ∆vjbR* strains were shown to be defective for survival within macrophages and rapidly cleared from the spleen in BALB/c mice [33]. The safety of the *∆vjbR* strains was further revealed by the absence of splenomegaly in inoculated mice [22]. Even at 2 weeks, when the bacterial load in the spleen was high, the mean spleen weights in Bm∆*vjbR* mice were 5-fold less than wild-type controls [33,128]. Remarkably, neither lethality nor osteoarticular disease was observed in severely immunodeficient interferon regulatory transcription factor 1 (IRF1) mice [114]. Therefore, the vaccine displayed unprecedented safety in preclinical animal trials. In contrast, the currently available animal vaccine strains S19 and Rev.1 induce splenomegaly in mice, an undesirable side effect for human vaccination [22,33]. Differences in survival and inflammatory responses exhibited by *B*. *melitensis* ∆*vjbR* strains are promising and warrant further evaluation in large animal and nonhuman primate models to develop an improved vaccine candidate for possible human use. In addition, an expanded analysis of delivery systems, including encapsulation of *B*. *melitensis* ∆*vjb*R or other LAV strains, is recommended [33]. For example, a recent study evaluated the protective and immunogenic potential of an alginate-encapsulated live attenuated *B*. *ovis* Δ*abcBA* vaccine [35]. Remarkably, this vaccine formulation prevented infection, bacterial shedding, and development of clinical changes and pathogenic lesions following challenge with wild-type *B*. *ovis* in rams [35]. An in vitro evaluation of the ∆*abcBA* strain in ovine monocyte-derived macrophages revealed defects in intracellular multiplication, trafficking, and *Brucella*-containing vacuole (BCV) maturation compared to wild-type infection [129]. Therefore, evaluation of candidate LAVs with alternative delivery systems and vaccine regimens (including dose and route) can potentially lead to LAVs with better efficacy and safety.

## Recent Developments with BTb Vaccines

The application of vaccines to address BTb has benefitted significantly from recent advances in vaccines for human TB. Live mycobacterial vaccines to replace BCG and subunit vaccines (virus vector or protein) to boost BCG have been tested. To date, field trials have demonstrated that BCG vaccination can protect cattle from natural exposure to *M*. *bovis* [130]. Similarly, calves vaccinated with novel *M*. *bovis* auxotrophs displayed reduced bacterial burden and pathology following challenge with virulent *M*. *bovis*. These data confirm the efficacy of LAVs in preventing infection with this pathogen. In contrast, subunit vaccine antigens, when delivered alone, have generally proven to be less efficacious than BCG in conferring protection to cattle from *M*. *bovis* (for review, see [73]). Moreover, culture filtrate proteins (CFP) from *M*. *bovis* [131] and DNA vaccines encoding *M*. *bovis* proteins have failed to provide similar levels of protection in cattle as BCG [132]. However, prime-boost combinations of BCG with DNA [133] or virus-vectored vaccines [134,135] have induced better protection than BCG vaccine alone, thereby demonstrating the utility of protective antigens in enhancing the immune protection initiated by LAVs [136]. In one example, boosting BCG vaccination with replication-deficient virus vectors encoding Ag85A induced strong cellular immunity, elevated interferon gamma (IFN-γ) responses, and enhanced protection with reduced pathogen loads following *M*. *bovis* challenge [134]. BCG strains overexpressing mycobacterial antigens may also be used as a delivery platform to increase vaccination efficacy in murine and guinea pig models [137]. Similarly, expression of the bacterial antigens *sodC* and *wboA* enhanced protection of *Brucella* RB51 vaccination against *B*. *suis* 1330 challenge [138,139]. Therefore, protective antigens can play an important role in prime-boost vaccination strategies.

Sequence analysis of BCG has provided information that can be exploited to develop novel candidate LAVs. For example, mutation of chromosomal regions of difference 1 (RD1) at the *cfp10-esat6* locus is responsible for loss of virulence [140]. *M*. *bovis ΔRD1* displayed a reduced number and severity of TB lesions as well as reduced bacterial burden in BCG vaccinates [36]. Another *M*. *bovis* mutant, *Δmce*2, exhibited greater immunological reactivity in response to tuberculin purified protein derivative (PPD) than BCG vaccinates [141]. Therefore, these strains may constitute potential vaccine candidates.

Although *B*. *melitensis* strains harboring mutations in the LuxR family member ∆*vjb*R have exhibited promise as LAVs for reducing brucellosis [22,114], similar targeting of this class of proteins in the development of BTb LAVs has not yet been thoroughly explored. Just recently, Mtb strains harboring deletions in the LuxR family transcription factor gene, Rv0195, of *M*. *tuberculosis* strain H37Rv were shown to decrease cell survival under hypoxic and reductive stress triggered by vitamin C [142]. Furthermore, Rv0195 deletion diminished bacterial virulence in human macrophage-like cells and resulted in reduced bacterial survival and pathogenicity in a C57BL/6 mouse infection model [142]. These studies raise the intriguing possibility that *M*. *bovis* strains harboring deletions in LuxR family members may provide a useful framework for the development of LAVs to address *M*. *bovis* infection.

## Perspective and Future Directions

As a result of their ability to elicit protective immune responses, LAVs against bacteria may represent a superior alternative to subunit, killed, or DNA vaccines. Although subunit vaccines have an advantage in perceived safety over live attenuated organisms, including viruses, their inability to penetrate deep lymphoid tissue results in a weak immune response lacking recall and frequently requiring the addition of toxic immune stimulants or attenuated vectors for delivery [143,144]. Additionally, bacteria and parasites engage multiple host factors and virulence mechanisms to invade cells or to evade or resist immune clearance. Using modern recombinant techniques, it is possible to irreversibly, temporally, or spatially attenuate the survival and/or transmissibility of some bacteria without restricting their immune potential. The one concern that prevents complete acceptance of LAVs is the potential for reversion to virulence, a concern fostered by the use of spontaneously occurring variants without knowledge of the extent of the genetic lesion, if any. In contrast to the use of such variants, modern techniques provide the opportunity to identify and remove species-defining genetic loci that are neither readily reacquired nor restore appropriate function. In addition, multiple loci may be removed, reducing the chance of reversion to infinitesimal levels.

We have argued that LAVs provide a compelling technology to control the neglected zoonotic diseases brucellosis and BTb; this is because of the better immune response they engender and a history of past successes in disease eradication [145]. We note, however, that LAV development across genera is not without potential disadvantages. For example, if the pathogen exerts significant immunomodulatory effects on the host, then the vaccine may also modulate aspects of the immune response and prevent the expression of resistance. Prior immunological exposure to a cross-reactive infectious or environmental agent may render the LAV ineffective because the vaccinating infection is terminated prematurely. LAVs may also raise safety concerns for immunocompromised individuals, which may preclude their use. The use of BCG in infants at risk of HIV exposure provides a salient example [146]. The removal of antibiotic resistance cassettes used for selection of targeted mutations in vaccine strains may be technically challenging in some organisms, which constitutes a potential impediment to licensure. Finally, the dependence of LAVs on a reliable cold chain may create challenges for delivery in resource-poor settings where neglected zoonotic diseases can ravage animal and human populations.

There are many exciting avenues for future research and testing of LAV vaccines. For example, the analyses of prime-boost strategies that pair promising *Brucella* subunit vaccines with corresponding candidate LAVs remain in their infancy. Efforts to genetically engineer a multivalent or universal *Brucella* vaccine that affords potent cross protection also constitutes an exciting area of future research. The development of encapsulation technologies to enhance the stability and reduce the requirement of cold chain storage for LAVs may also transform the feasibility of large-scale vaccination efforts [33,147]. Engineering LAVs to protect against multiple pathogens, including *M*. *bovis* and *Brucella*, provides an exciting avenue of future investigation. Finally, new advances in genetics, bioinformatics, and pharmaceutical technology may provide avenues for addressing these challenges and thereby promote development of next-generation vaccines for addressing neglected zoonotic diseases of global consequence.

**Key Learning Points**

- LAVs against brucellosis and Btb display greater efficacy and/or safety in various model systems than competing subunit vaccine platforms against these diseases.
- Some candidate *Brucella* LAVs have demonstrated excellent efficacy and safety in preclinical studies.
- The potential for reversion to virulence of LAVs has been partly addressed by the use of modern techniques that remove species-defining genetic loci that are neither readily reacquired nor restore function, and the removal of multiple loci reduces the risk of reversion to infinitesimal levels.
- Evaluation of prime-boost strategies, a multivalent or universal *Brucella* vaccine, encapsulation technologies to deliver *Brucella* LAVs, and engineering LAVs to protect against multiple pathogens, including *M*. *bovis* and *Brucella*, provides exciting avenues of future investigation.
- High-throughput approaches for generating and screening banks of mutant bacteria, including the TraSH system and RNA-guided gene editing using CRISPR-Cas technology, provide exciting areas for future investigation.

**Top Five Papers in the Field**

1. Arenas-Gamboa AM, Ficht TA, Kahl-McDonagh MM, Gomez G, Rice-Ficht AC. The *Brucella abortus* S19 Δ*vjbR* live vaccine candidate is safer than S19 and confers protection against wild-type challenge in BALB/c mice when delivered in a sustained-release vehicle. Infect Immun. 2009;77(2): 877–84.
2. Silva APC, Macêdo AA, Costa LF, Rocha CE, Garcia LNN, Farias JRD, et al. Encapsulated *Brucella ovis* lacking a putative ATP-binding cassette transporter (Δ*abcBA*) protects against wild type *Brucella ovis* in rams. PLoS ONE. 2015;10(8): e0136865.
3. Vemulapalli R, He Y, Buccolo LS, Boyle SM, Sriranganathan N, Schurig GG. Complementation of *Brucella abortus* RB51 with a functional *wboA* Gene results in O-antigen synthesis and enhanced vaccine efficacy but no change in rough phenotype and attenuation. Infect Immun. 2000;68(7): 3927–32.
4. Pym AS, Brodin P, Brosch R, Huerre M, Cole ST. Loss of RD1 contributed to the attenuation of the live tuberculosis vaccines *Mycobacterium bovis* BCG and *Mycobacterium microti*. Mol Microbiol. 2002;46(3):709–17.
5. Skinner MA, Ramsay AJ, Buchan GS, Keen DL, Ranasinghe C, Slobbe L, et al. A DNA prime-live vaccine boost strategy in mice can augment IFN-γ responses to mycobacterial antigens but does not increase the protective efficacy of two attenuated strains of *Mycobacterium bovis* against bovine tuberculosis. Immunology. 2003;108(4):548–55.
